# Danshen (*Salvia miltiorrhiza*) restricts MD2/TLR4‐MyD88 complex formation and signalling in acute myocardial infarction‐induced heart failure

**DOI:** 10.1111/jcmm.15688

**Published:** 2020-08-05

**Authors:** Xiaoping Wang, Dongqing Guo, Weili Li, Qian Zhang, Yanyan Jiang, Qiyan Wang, Chun Li, Qi Qiu, Yong Wang

**Affiliations:** ^1^ School of Life Science Beijing University of Chinese Medicine Beijing China; ^2^ School of Traditional Chinese Medicine Beijing University of Chinese Medicine Beijing China; ^3^ Department of Pharmacy Beijing Anzhen Hospital Capital Medical University Beijing China; ^4^ School of Chinese Materia Medica Beijing University of Chinese Medicine Beijing China

**Keywords:** acute myocardial infarction, Danshen, heart failure, inflammation, TLR4

## Abstract

Heart failure (HF) represents a major public health burden. Inflammation has been shown to be a critical factor in the progression of HF, regardless of the aetiology. Disappointingly, the majority of clinical trials targeting aspects of inflammation in patients with HF have been largely negative. Many clinical researches demonstrate that danshen has a good efficacy on HF, and however, whether danshen exerts anti‐inflammatory effects against HF remains unclear. In our study, the employment of a water extracted and alcohol precipitated of danshen extract attenuated cardiac dysfunction and inflammation response in acute myocardial infarction‐induced HF rats. Transcriptome technique and validation results revealed that TLR4 signalling pathway was involved in the anti‐inflammation effects of danshen. In vitro, danshen reduced the release of inflammatory mediators in LPS‐stimulated RAW264.7 macrophage cells. Besides, the LPS‐stimulated macrophage conditioned media was applied to induce cardiac H9C2 cells injury, which could be attenuated by danshen. Furtherly, knock‐down and overexpression of TLR4 were utilized to confirm that danshen ameliorated inflammatory injury via MyD88‐dependent TLR4‐TRAF6‐NF‐κB signalling pathway in cardiomyocytes. Furthermore, by utilizing co‐immunoprecipitation, danshen was proved to suppress MD2/TLR4 complex formation and MyD88 recruitment. In conclusion, our results demonstrated that danshen ameliorates inflammatory injury by controlling MD2/TLR4‐MyD88 complex formation and TLR4‐TRAF6‐NF‐κB signalling pathway in acute myocardial infarction‐induced HF.

## 1 INTRODUCTION

As a chronic, progressing and ultimately debilitating syndrome, heart failure (HF) is currently recognized as a major and escalating public health concern worldwide.^1^ In particular, patients surviving from acute myocardial infarction (AMI) are at a high risk of developing HF, and incidence of HF among patients hospitalized for an AMI differs among studies, ranging from 14% to 36% in the last three decades, indicating that current therapeutic strategies still miss one or more effective pharmacologic interventions.^2^ Therefore, developing a new therapy to prevent AMI‐induced HF is urgent.

Researches suggested that ischaemic injury initiates an excessive or persistent inflammation response that leads to further cardiac tissue destruction, cardiac dysfunction and eventually to HF.^3^, ^4^ Recognizing the regulation of therapeutic drugs on the molecular mechanism of inflammation is crucial for controlling HF progression. Toll‐like receptors (TLRs), as primary receptors of innate immunity, could initiate innate immune defence by interacting with pro‐inflammatory pathways, in turn leading to the development and deterioration of inflammatory diseases.^5^, ^6^ It is reported that following myocardial infarction, necrotic cardiomyocytes and damaged extracellular matrix release danger signals, binding a variety of TLRs in various tissues and cells of cardiovascular system which aggravate the inflammatory response during HF progress, suggesting the potential use of TLRs in target therapy.^5^, ^7^, ^8^ In the heart, one of the most highly expressed and the most studied in the context of myocardial injury is Toll‐like receptor 4 (TLR4).^4^ TLR4 activation requires complex formation with myeloid differentiation protein 2 (MD2). Next, TLR4/MD2 engages with the myeloid differentiation factor 88 adaptor protein (MyD88) directly to trigger receptor complex interactions with TNF receptor‐associated factor 6 (TRAF6) and transforming growth factor‐activated kinase 1 (TAK1).^9^ The signalling cascade ultimately activates the phosphorylation, ubiquitylation and degradation of inhibitor of NF‐κB (IκB), which further triggers the nuclear transportation of factor‐kapa B (NF‐κB) to induce a broad array of pro‐inflammatory mediators.^5^ Moreover, p38 MAPK, as a TAK1 downstream kinase, has recently been implied in inflammation.^10^ Thus, focusing on TLR4/MD2‐MyD88 complex formation and signalling axis is gaining increasing interest as a potential therapeutic or preventive strategy for the treatment of AMI‐induced HF. The importance of inflammatory response to stressed cardiomyocytes is well accepted; however, the definite inflammatory mechanisms in cardiomyocytes are not well understood. This research elucidated that to some content, stressed cardiomyocytes may play the role of ‘inflammatory cells’.

*Salvia miltiorrhiza Bunge*, also known as danshen (DS) in Chinese, is a star herb in the Traditional Chinese Medicine (TCM) and is demonstrated to exhibit various pharmacological activities, such as anti‐inflammation, anti‐atherogenesis, antioxidation, anti‐diabetes and anti‐tumour.^11^ The herb has been clinically used for more than 2000 years to treat numerous ailments, especially cardiovascular diseases and has been officially recorded in the Chinese pharmacopoeia since 1953. In the last 2 decades, 39 clinical trials comprised a total of 2431 patients have been conducted where danshen was used alone or in combination with other herbs to treat cardiovascular diseases.^12^ Among them, 2 trials used danshen alone showed that danshen has a potential protective effect on the development of cardiovascular disease.^13^, ^14^ Impressively, a randomized controlled trial demonstrated that danshen was beneficial to the diabetic patients with chronic heart disease.^13^ Some other clinical trials showed that danshen could also significantly reduce the ventricular hypertrophy index, improve the function of ventricular myocardium and regulate the diastolic function of the ventricle.^12^ In general, the results from the clinical trials have demonstrated that danshen is promising for the management of heart failure. However, the underlying mechanism is still blurred.

In the current study, the highly reproducible rat model of left anterior descending (LAD) ligation is used to mimic myocardial infarction and induce heart failure and evaluate the cardioprotective effects of danshen. Importantly, transcriptome technique was applied to explore potential differential inflammation‐related genes and pathways. Our studies will elucidate the anti‐inflammation mechanisms of danshen and provide alternative therapeutic approaches for the treatment of HF.

## 2 MATERIALS AND METHODS

### 2.1 Materials

#### 2.1.1 Chemicals

4% paraformaldehyde and 0.9% saline were purchased from Beijing Applygen Technology Inc (Beijing, China). Foetal bovine serum (FBS), Dulbecco's modified Eagle's medium (DMEM), streptomycin, penicillin, phosphate buffered solution (PBS), DAPI, sterile ultrapure water and LPS were purchased from Beijing BioDee Biotechnology Co., Ltd. (Beijing, China). TLR4 pcDNA 3.1, LipoFiter 3.0, TLR4 siRNA and RNAfit (HB‐RF‐1000) were purchased from Hanbio Technology Co., Ltd. (Shanghai, China). All other chemicals were purchased from formal commercial sources.

#### 2.1.2 Danshen preparation and quality assessment

We applied the preparing procedures of water extraction and alcohol precipitation for danshen samples. To ensure stable quality of danshen samples, we established fingerprints by HPLC‐PDA for discrimination of samples. The standard of quality control referred to China Pharmacopoeia. Briefly, danshen was prepared using the following procedure: accurately weighed lyophilized powder (0.2 g) was thoroughly suspended in 10 volumes of 5% aqueous ACN; followed by centrifugation (9600 rpm) for 10 mins, then, the supernatant was filtered through a 0.22 μm membrane before LC‐MS analysis; finally, the injection volume and other LC conditions were set.

### 2.2 Establishment of AMI rat model and pharmacological treatments

SPF‐grade Sprague Dawley (SD) rats (260 ± 10 g) were purchased from Beijing SPF Biotechnology Co., Ltd., China (Beijing, China). All rats were housed at 23 ± 1°C, with proper humidity and lighting (12 hours light/dark cycle). Forty rats were randomly divided into six groups as below: sham group, model group, DS‐L; DS‐M; DS‐H (with dosages of 3.75; 7.5; 15 μg/kg) group and positive drug trimetazidine (TMZ, with a dosage of 10 mg/kg) group, respectively. Danshen's dosage was given according to its percentage contained in Danqi pill, and the dosage of Danqi pill (Series: 6128006; Beijing Tongren tang, China,) was referring to clinical application and built by the equivalent conversion between rat and people. In our previous study, ligation of left anterior descending (LAD) coronary artery‐induced AMI rat model was described detailly.^15^ Drugs were orally and daily administered for 28 days starting on first day after surgery. Since trimetazidine is conventionally used for cardiovascular diseases especially left ventricular (LV) dysfunction, without relevant side effects, we utilized it as positive drug.^16^ The sham group and the model group received the same volume of 0.9% saline. All animal procedures were approved by Beijing University of Chinese Medicine’ Animal Care Committee and confirmed to the guidelines from directive 2010/63/EU of the European Parliament on the protection of animals used for scientific purposes. The approved code for our animal experiment is ‘BUCM‐4‐2018101504‐4068’. At the end of experiment, all rats were anaesthetized by isoflurane inhalation 2% and finally euthanized by cervical dislocation.

### 2.3 Echocardiographic assessment of cardiac functions

After 28 days’ consecutive administration, cardiac function was examined by Transthoracic Echocardiography (Vevo TM 2100; Visual Sonics, Canada). LV end‐diastolic diameter (LVEDD) and LV end‐systolic diameter (LVESD) were assessed for at least three consecutive cardiac cycles. Per cent fractional shortening (FS) and ejection fraction (EF) were calculated.

### 2.4 Histological examination and Masson's trichrome staining

The hearts were immersed in 4% paraformaldehyde for at least 24 hours, then embedded in paraffin and sectioned into 4 μm slices to be stained with haematoxylin‐eosin (HE) or Masson's trichrome staining. The images were obtained by optical microscope at 400 x magnification. The semiquantitative results of inflammatory cells and collagen volume fraction (CVF) were presented by using ImageJ software (https://imagej.nih.gov/ij/).

### 2.5 Detection of biomarkers

B‐type natriuretic peptide (BNP) is one of the gold standard biomarkers in determining the diagnosis and prognosis of HF.^17^ Tumour necrosis factor‐α (TNF‐α) and interleukin‐1β (IL‐1β) have been demonstrated as inflammatory biomarkers in chronic and acute HF patients.^18^ LDH locates in the cytoplasm of cardiomyocytes in normal state. The release of LDH into blood is considered diagnostic biomarker of AMI‐induced HF.^19^ Levels of BNP, TNF, IL‐1β, NO and lactate dehydrogenase (LDH) in cardiac tissue or cell supernatant were detected by following the instructions of commercially available kits (Nanjing Jiancheng, China). The content was expressed as pg/mL or μM.

### 2.6 Transcriptome sequencing (RNA‐seq) analysis

#### 2.6.1 RNA Preparation

Total RNA of the cardiac tissues was extracted using TRIzol Reagent® (Invitrogen, Carlsbad, CA) according to the manufacturer's instruction. Extracted RNA was digested with DNase to remove contaminating genomic DNA, and the quality of RNA was evaluated by RNA Nano 6000 Assay Kit of the Agilent Bioanalyzer 2100 system (Agilent Technologies, CA, USA). RNA was purified using poly‐T oligo‐attached magnetic beads, then reverse transcribed into cDNA. After cDNA was ligated with adaptors, PCR amplification was performed using Phusion High‐Fidelity DNA polymerase, Universal PCR primers and Index (X) Primer and build the library of each sample. The library was sequenced on Illumina Hiseq4000 platform and 150bp paired‐end reads. Quality control and alignment were performed with rat reference sequences. Subsequently, read counts of each gene were computed as raw gene expression.

#### 2.6.2 Differential Expression Analysis and KEGG pathway enrichment

Using R package ‘DESeq2’, differentially expressed genes (DEG) were identified with a *P*‐value < 0.05 and |log2(foldchange)|> log2(1.5) in R version 3.5.1 software. Furthermore, KEGG pathway enrichment was performed using R package ‘clusterProfiler’ with *P* < 0.05 (R version 3.5.1 software).

### 2.7 Real‐time quantitative PCR to verify the results of the Transcriptome

The sequences of primers used for fluorescent quantitative PCR are shown in Table 1. TRIzol Reagent (Gibco‐BRL, Paisley, UK) was used to extract the total RNA from each sample. The NanoDrop 2000 (Thermo Scientific, USA) was utilized for measuring the RNA concentration and purity, and then, the Revert Aid First Strand cDNA Synthesis kit (Fermentas, LT) was used to reverse transcribe the cDNAs. The SYBR Select Master MIX (Applied Biosystems, USA) was applied to detect the expression of the target genes through using fluorescent quantitative PCR. Samples were amplified by 95°C for two minutes, 40 cycles of 95°C for 30 seconds, 95°C for five seconds, 60°C for five seconds and 72°C for 10 minutes. Finally, the 2−△△CT method was used to analyse the gene expression in each sample.

**Table 1:** Nucleotide sequences of primers used in real‐time PCR

Gene	Primers
Tlr4	ATTGTATCGCCTTCTTAGCA
	CTTCTTGTTCTTCCTCTGATG
Tlr2	GCTGTGGTATCTGAGAATGA
	GAATCCTGCTCGCTGTAG
Tlr9	TGGACCTAAGCGAGAACT
	GAGCAAGCGGAAGAAGAT
Cd86	AATGAGTATGGCGACAACA
	AGATAGGCTGATGGAGACA
Cd80	TGTCCAAGTCGGTGAGAG
	TGCCAGTAGATTCGGTGTA
GAPDH	GGATACTGAGAGCAAGAGAGA
	TTATGGGGTCTGGGATGGAA
Tlr8	TCAGAGATGGAAGAGTGTCA
	TGGAGGTGGTAAGGAATGT
Irf5	AGGAGGAAGAGGAAGATGAA
	CCAGGTAGCACAGGTTCT
Spp1	CCATCTCAGAAGCAGAATCT
	CATCGTCGTCGTCATCAT

### 2.8 Cell culture

RAW 264.7 macrophages and rat myocardial H9C2 cell used in the present study were purchased from China Infrastructure of Cell Line Resources and were both cultured at 37 **°C** under 5% CO2 humidified atmosphere, with DMEM supplemented with FBS (10%), Penicillin (100 U/mL) and Streptomycin (100 μg/mL). To screen the non‐toxic concentrations of danshen, H9C2 and RAW264.7 cells were seeded onto 96‐well plates and incubated with danshen (100 ~ 1200μg/mL) for 24 hours. Then, Cell Counting Kit‐8 (CCK8, Dojindo, Kumamoto, Japan) was utilized to assess the cell viabilities by measuring absorbance at 450 nm under Microplate reader (perkin‐elmer, Waltham, MA and United States).

To evaluate the effects of danshen on LPS‐induced macrophages, RAW264.7 cells were exposed to LPS (1 μg/mL) for 24 hours with/without danshen. Cell supernatants for conditioned media (CM) were collected for further experiments. To investigate the effects of danshen on CM‐stimulated cardiomyocytes, H9C2 cells were pre‐treated with danshen for 6 hours and then stimulated with CM (with/without danshen) for 24 hours.

### 2.9 Knock‐down of TLR4 with siRNA

TLR4 expression was knocked down using TLR4 siRNA (Hanbio Technology, Shanghai, China) according to the manufacturer's instructions. The sequence (5’‐3’) is GGAUCAGAAUCUCAGCAAAdTdT; UUUGCUGAGAUUCUGAUCCdTdT. Briefly, H9C2 cells were incubated with 500 μL RNAfit and 10 μL siRNA for 6 hours, and then, the medium was replaced by DMEM (2% FBS; antibiotics‐free) for another 18 hours. Cells transfected with non‐specific scramble siRNA (NC siRNA) were used as controls. RNA was extracted to check the knock‐down efficiency by PCR (Figure 5D). Cells were subjected to experiment at 40 hours after transfection.

#### 2.9.1 Overexpression of TLR4 with pcDNA3.1

TLR4 was overexpressed by using TLR4 pcDNA3.1 (Hanbio Technology, Shanghai, China) according to the manufacturer's instructions. Briefly, H9C2 cells were plated into 6‐well plates for 24 hours. The medium was replaced with DMEM (antibiotic‐free) two hours before transfection. Then, cells were transfected with 1 μg plasmid (pcDNA3.1‐TLR4 or empty vector pcDNA3.1) and 25 μL LipoFiter 3.0/well. After 24 hours, cells were subjected to experiment.

#### 2.9.2 Immunofluorescence

Firstly, the paraffin‐embedded sections were inactivated with 0.3% hydrogen peroxide for 15 mins and blocked with goat serum for 10 minutes at room temperature. Then, sections were incubated with anti‐rabbit IgG polyclonal for 1 hour at room temperature in the dark. Followed by incubation with TLR4 antibody at 4°C overnight, DAPI staining was carried out at room temperature for 5 minutes in the dark. Finally, sections were washed and fixed with antifade mounting medium. The optical microscope was used for photographing at 400 × magnification (Leica Microsystems GmbH).

Cells were grown on a laser confocal dish for different treatments and then fixed with 4% paraformaldehyde for 15 mins, followed by permeabilization (0.5% Triton x‐100 in 0.1 mol/L PBS) for 20 mins and blocking with goat serum for 1 hour. Thereafter, cells were stained with NF‐κB antibody overnight at 4°C and incubated with anti‐rabbit IgG antibody in the dark at room temperature for 1 hour, followed by washing three times with PBS and counterstaining with DAPI (10 μg/mL) for 30 minutes.

#### 2.9.3 Co‐immunoprecipitation and Western blots analysis

Prepared cell extracts from different groups were incubated with 1 μg anti‐MD2 (R&D system) or anti‐TLR4 (ab8376, Abcam, UK) for 1 hour at 4°C, and immunoprecipitation was made with protein A Agarose Beads (9863T, CST, USA) at 4°C overnight. Finally, samples were immunoblotted for detection of TLR4 or MyD88 as co‐precipitated protein.

Myocardial tissues or cells were homogenized with ice‐cold RIPA lysate (Beijing Applygen Technology Inc, China) containing 1% protease inhibitor and 2% protein phosphatase inhibitor (Beijing Applygen Technology Inc, China) and then centrifuged at 12 000 g for 10 minutes at 4°C. Bicinchoninic Acid (BCA) Protein Assay Kit (Beijing Applygen Technology Inc, China) was used to measure the protein concentration. Quantitative samples were separated on 10% SDS‐PAGE gels and transferred to polyvinylidene fluoride (PVDF) membranes. The membranes were blocked with 5% skim milk in 1 x Tris‐buffered saline for 2 hours, followed by incubation with the primary antibodies at 4°C overnight and secondary antibody at room temperature for 1 hour. The membranes were exposed to ECL (ECL Plus Western blotting Detection Reagent, GE Healthcare, United States) in the darkroom for about 10 seconds. Density of bands was quantified by ImageJ. The antibodies we used are listed in Table 2.

**Table 2:** Primary antibodies used in Western blot experiment

Protein	Primary antibody	Concentration
TLR4 MyD88 TRAF6 IκBα p‐IκBα MAPK p‐MAPK NF‐κB p‐NF‐κB GAPDH	TLR4 Antibody, 19811‐1‐AP, Proteintech Anti‐MyD88, ab2064, Abcam Anti‐TRAF6, ab227560, Abcam Ant‐IKBα, ab32518, Abcam Phospho‐IκBα (Ser32), 2859, CST p38 MAPK, 2387, CST Phospho‐p38 MAPK (Thr180/Tyr182), 9215, CST Anti‐NF‐κB p65, ab16502, Abcam Anti‐NF‐κB p65 (phospho S529), ab97726, Abcam Anti‐GAPDH, ab8246, Abcam	1:1000 1:1000 1:1000 1:1000 1:1000 1:1000 1:1000 1:1000 1:1000 1:2000

#### 2.9.4 Data analysis

Statistical analyses were performed on GraphPad Prism software 6.0 (San Diego, CA, USA). All results are presented as the means ± SD. Comparisons between two groups were performed with the unpaired two‐tailed t test. Multiple comparisons were determined using ANOVA followed by Bonferroni‐corrected post hoc test. The difference was considered statistically significant when *P* < 0.05.

## 3 RESULTS

### 3.1 Formatting of mathematical components

**Figure 1:**
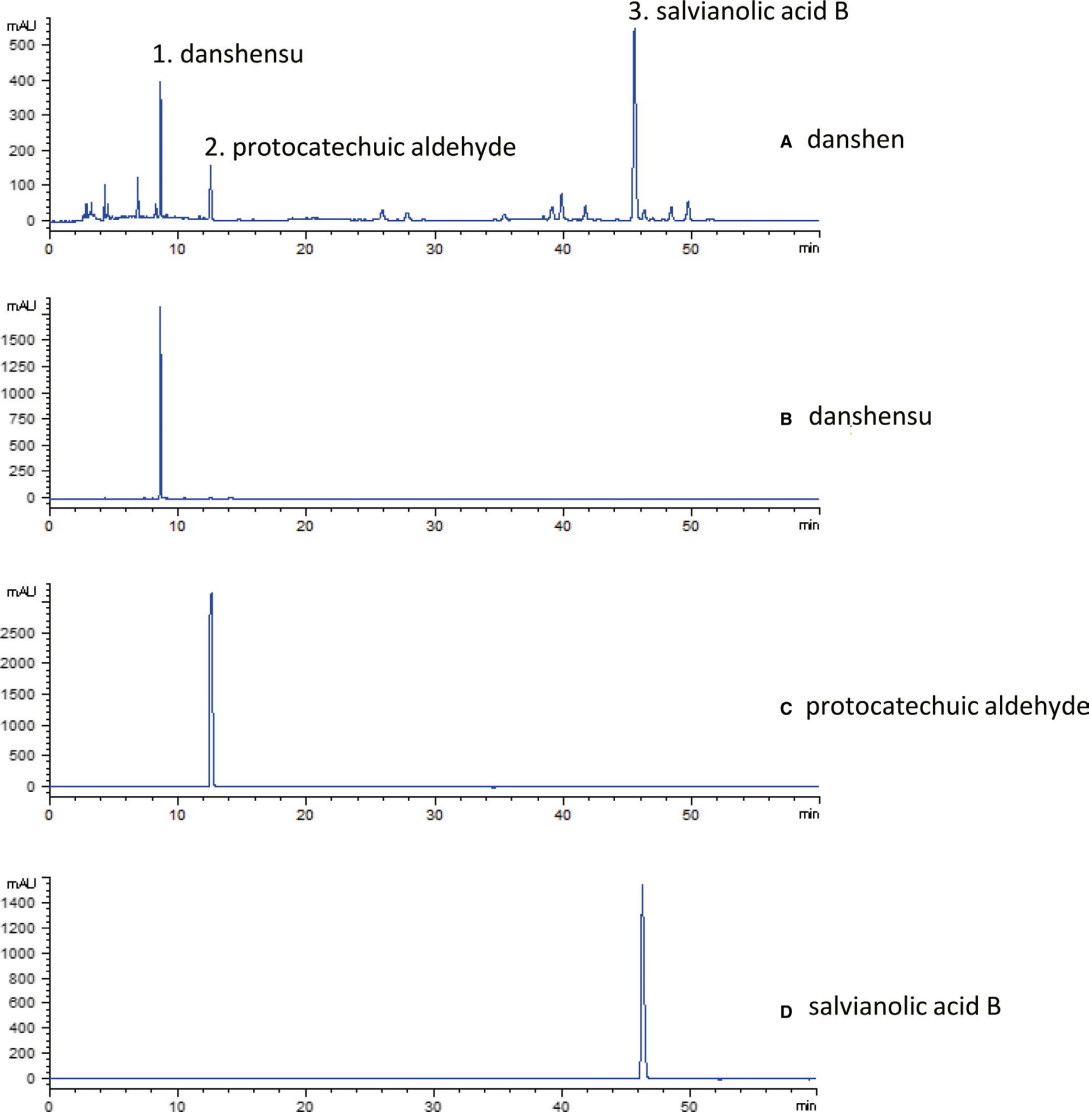
Qualitative analysis on grinding powder of danshen. a, danshen; b/1, danshensu; c/2, protocatechuic aldehyde; d/3, salvianolic acid B

HPLC‐PDA was used for the components analysis and 17 characteristic peaks in the danshen‐fingerprint chromatograms were identified (Figure 1). By comparing with HPLC retention time of standard danshen according to China Pharmacopoeia (Ministry of Health of the People's Republic of China Pharmacopoeia Committee, 2010), we identified three dominating peaks as danshensu, protocatechuic aldehyde and salvianolic acid B. HPLC‐PDA chromatograms numbered from 1 to 3 represent danshensu, protocatechuic aldehyde and salvianolic acid B.

### 3.2 Danshen attenuates left ventricular (LV) dysfunction and reduces inflammation response in HF post‐AMI rats

**Figure 2:**
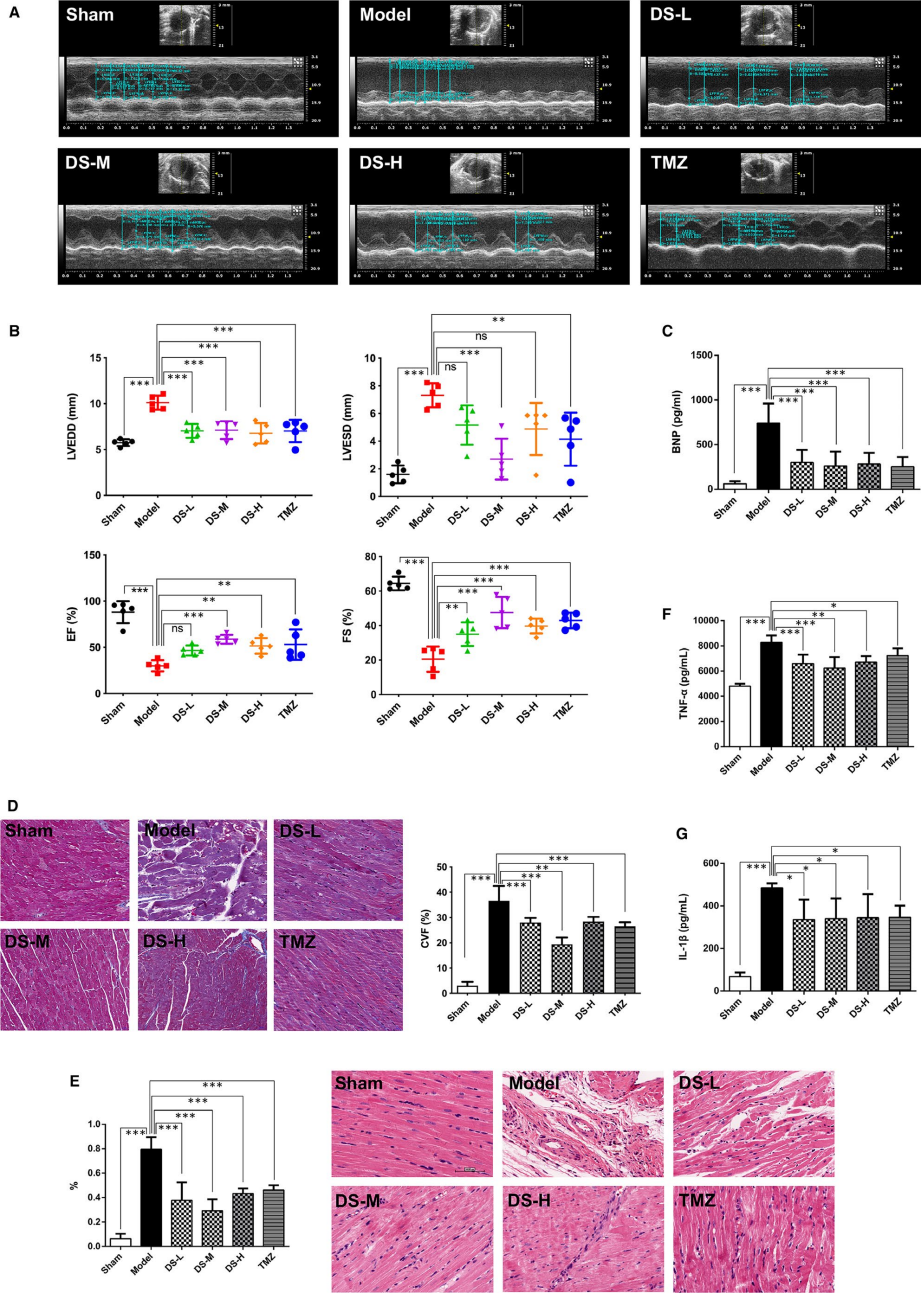
Danshen is shown to improve heart function and alleviate pathological changes in rats. (A) M‐mode echocardiographic images obtained from Sham, Mode, DS‐L, DS‐M, DS‐H and TMZ groups 28 days post‐AMI. (B) Analysis of LV end‐diastolic diameter (LVEDD) and LV end‐systolic diameter (LVESD); and per cent fractional shortening (FS) and ejection fraction (EF) calculations. (C) Levels of BNP in plasma from different groups. (D) Representative heart photomicrographs of Masson trichrome staining and semiquantitative results of collagen volume fraction (CVF) from different groups. (E) Representative heart photomicrographs of HE staining from different groups and the inflammatory cells were quantified (%) in the HE staining. Scale bar = 20 μm. Levels of cytokines including TNF‐α (F) and IL‐1β (G) in cardiac tissue from different groups. N = 5 per group, *P < .05, **P < .01, ***P < .01, ns means *P* > .05, that is no significance

LV function was detected by echocardiography. M‐mode tracings were analysed at 28 days post‐AMI (Figure 2A). AMI increased LVEDD and LVESD and decreased EF and FS (Figure 2B). Danshen treatment with different dosages improved cardiac dysfunction with significantly lowered LVEDD and LVESD, and increased EF and FS compared to the model group (Figure 2B). Besides, danshen treatment significantly reduces the release of BNP (Figure 2C). On Masson's trichrome staining results, the model group showed accumulation of collagen fibres (Figure 2D). On HE staining results, heart showed normal myocardial morphology in the sham group, whereas some myocardial distortion and mononuclear cell infiltration were noted in the model group (Figure 2E). Treatment with danshen in different dosages protected hearts against collagen deposition, pathological changes and inflammatory cell infiltration caused by AMI (Figure 2D,E). In addition, levels of TNF‐α and IL‐1β in cardiac tissue were higher in rats of the model group (Figure 2F,G), whereas danshen treatment with different dosages reversed these changes, respectively (Figure 2F,G) suggesting a definite anti‐inflammatory effect on HF which is consistent with the clinical results. Collectively, danshen treatment showed a good cardioprotective effect against HF post‐AMI rats. Trimetazidine had the similar effects as danshen (Figure 2A‐G).

### 3.3 Transcriptome results and validation of the key genes in TLR signalling pathway

#### 3.3.1 Differential expression analysis

In order to study transcriptional regulatory networks, transcriptome technique was applied. The results of differential expression analysis are shown in Table S1. There are 843 differentially expressed genes between the DS (15 μg/kg) group and the model group including 387 up‐regulated genes and 456 down‐regulated genes. The changes in expression of these genes indicated the broad effects of danshen treatment on HF post‐AMI.

#### 3.3.2 Quantitative pathway analysis

**Figure 3:**
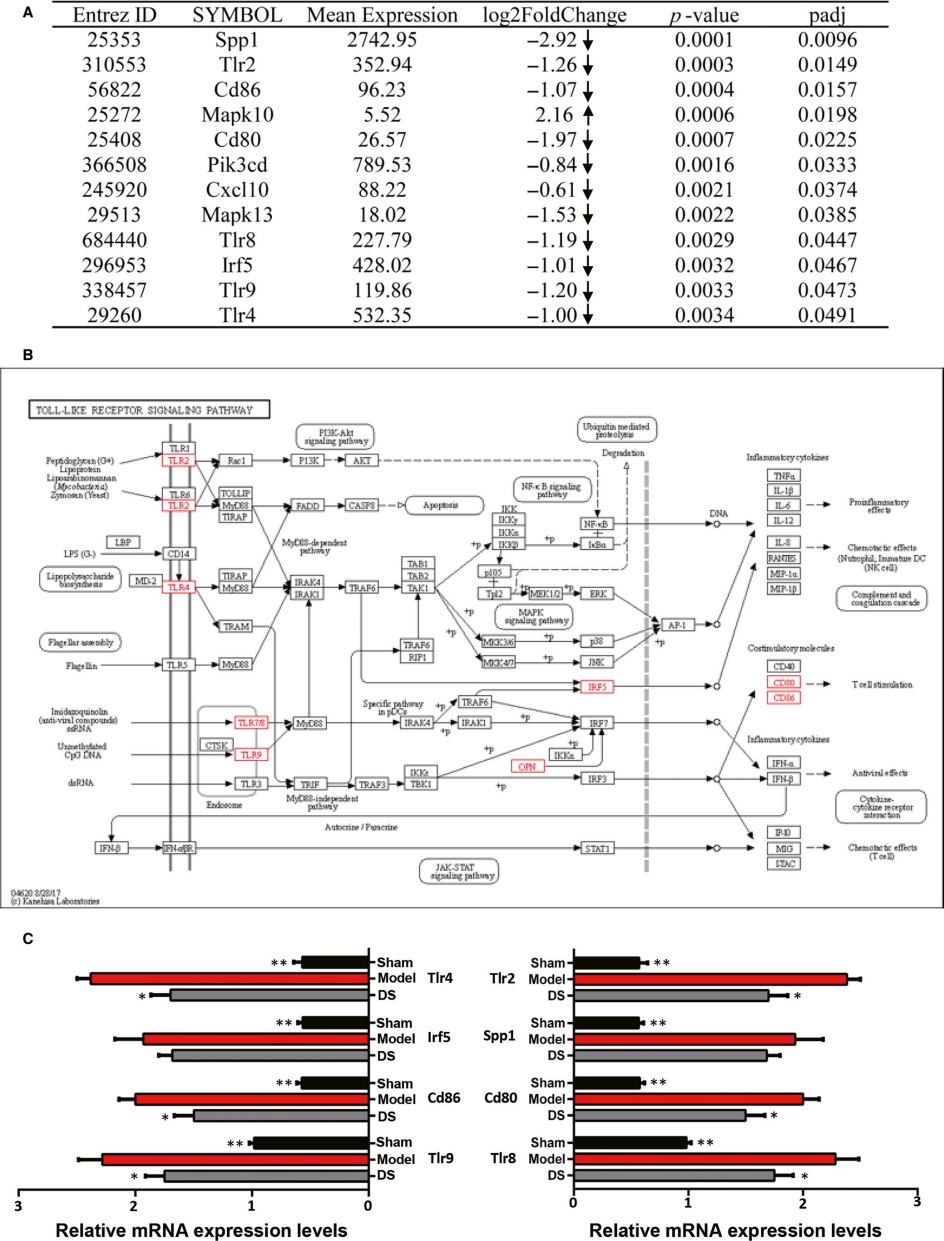
Danshen is shown to regulate the TLR signalling pathway. (A) The differentially expressed genes between the DS (15 μg/kg) group and the model group in the TLR signalling pathway. The arrows represent the up‐regulated or down‐regulated expression of gene. (B) Toll‐like receptor signalling pathway—reference pathway in KEGG. (C) Gene expressions of Tlr4, Cd86, Tlr9, Tlr2, Cd80, Tlr8, Irf5 and Spp1 were validated by using real‐time fluorescent quantitative PCR. Values were calculated by transforming the estimated marginal means from the RM‐ANOVA with a fold change = 2−DDCT. N = 5 per group, *P < .05, **P < .01

A total of 70 pathways are enriched with the differentially expressed genes between the DS (15 μg/kg) group and the model group. Pathway enrichment results are listed in Table S2. The analysis of pathway enrichment demonstrated that the TLR signalling pathway, the chemokine signalling pathway and the natural killer cell‐mediated cytotoxicity were altered, which are closely related to inflammatory responses. The differentially expressed genes in the TLR signalling pathways are shown in Figure 3A.

#### 3.3.3 Validation of the key genes in TLR signalling pathway

Based on transcriptome results, we validated eight genes, which are considered to be critical in TLR signalling pathway (Figure 3B). Figure 3C showed gene expression results for the different treatment groups from PCR analysis. Based on PCR results, the expressions of the Tlr4, Irf5, Cd86, Tlr9, Tlr2, Spp1, Cd80 and Tlr8 genes significantly increased in the model group when compared with the sham group (*P* < .01 vs Sham group). Whereas compared with the model group, the expressions of Tlr4, Cd86, Tlr9, Tlr2, Cd80, Tlr8 genes and Irf5, Spp1 genes in the DS (15 μg/kg) group decreased, respectively.

### 3.4 Danshen regulates the expressions of key signalling molecules of TLR4 signalling pathway in rats

**Figure 4:**
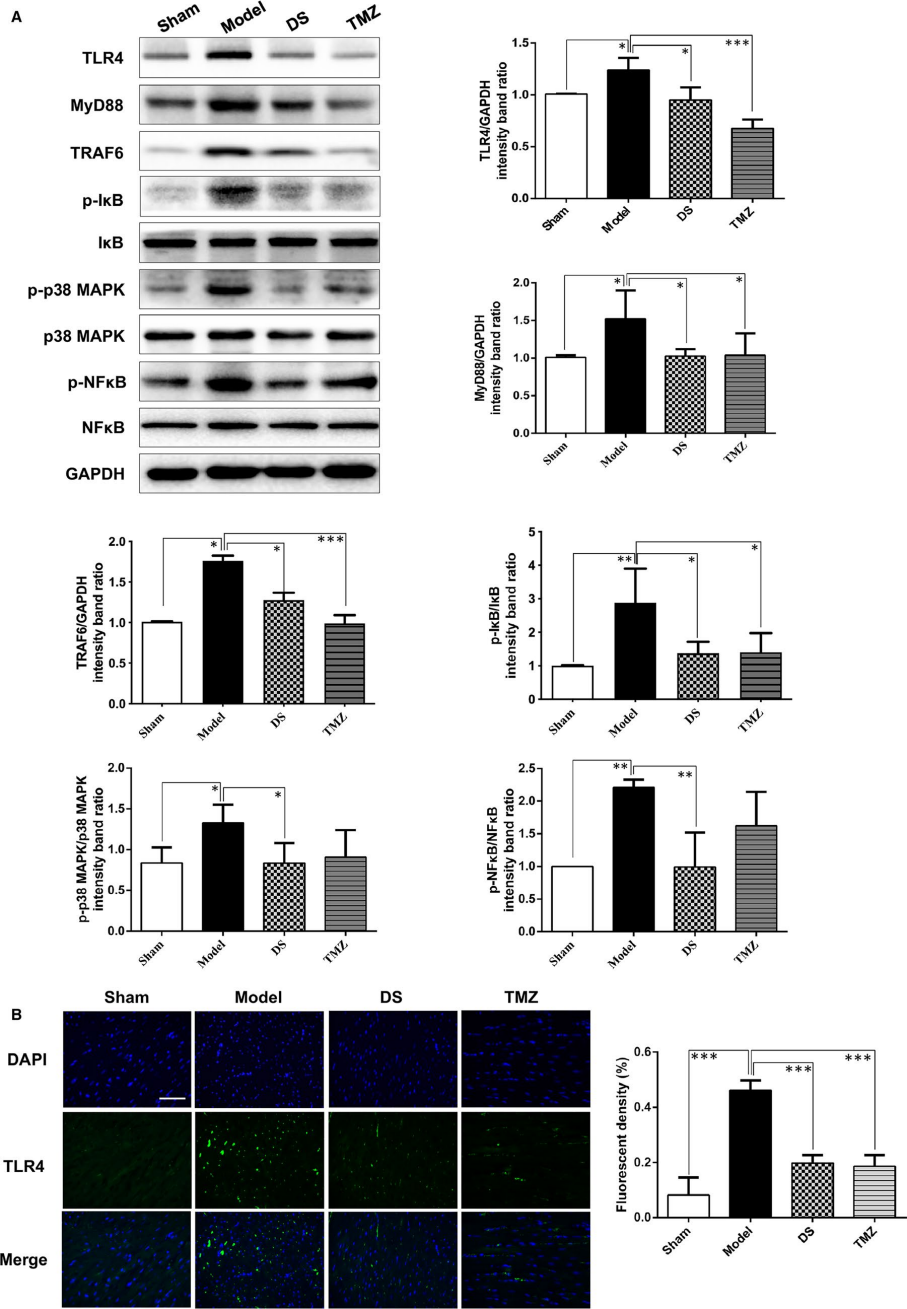
Danshen is shown to regulate critical signalling molecules of TLR4 signalling pathway. (A) Western blots images and analysis of TLR4, MyD88, TRAF6, p‐IκB, p‐p38 MAPK and p‐NF‐κB. N = 3. (B) IHC and quantitative result of TLR4 in different treatment groups, scale bar = 20 μm, N = 5. *P < .05, **P < .01, **P < .01

Effects of danshen on TLR4 signalling pathway‐related proteins were furtherly investigated by Western blots. The results showed that compared with the sham group, levels of TLR4, MyD88, TRAF6, p‐p38 MAPK (Figure 4A) and p‐IκB, p‐NF‐κB (Figure 4A) were increased in the model group, while danshen treatment (15 μg/kg) down‐regulated the expressions of these signalling molecules, respectively (Figure 4A). IHC of TLR4 was assessed, and the result was consistent with Western blots (Figure 4B). Collectively, these data indicated that danshen exerted marked effects on TLR4 signalling pathway.

### 3.5 Danshen inhibits macrophage conditioned media‐induced inflammation response in H9C2 cells

**Figure 5:**
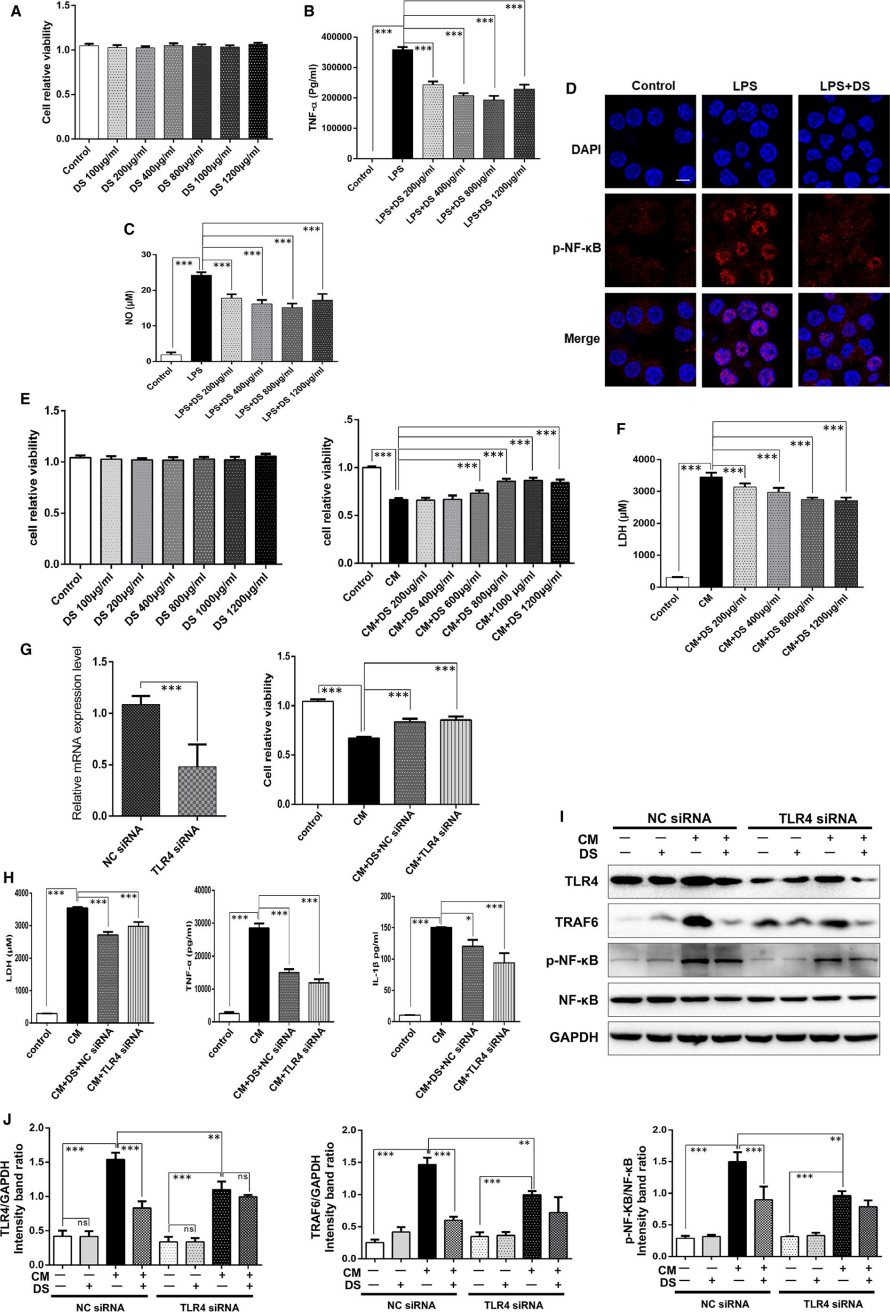
Danshen is shown to protect cardiomyocytes against CM‐induced inflammation. (A) The CCK8 assay showed that danshen treatment for 24 hours exerted no cytotoxic effect on macrophages below 1200 μg/mL, N = 12. Levels of TNF‐α (B) and NO (C) in macrophage supernatants were assessed by Elisa assay, N = 5. (D) Immunofluorescence images of p‐NF‐κB were shown from different groups in macrophage, scale bar = 50 μm, N = 12. (E) The CCK8 assay showed that danshen demonstrated no cytotoxic effect on cardiomyocytes below 1200 μg/ml and showed the effective concentrations of danshen, N = 12. (F) The release of LDH in H9C2 cell supernatants was detected by ELISA assay, N = 12. (G) RNA was extracted to check the knock‐down efficiency by PCR, N = 12. TLR4 siRNA increased the cell viability (G) and reduced the release of LDH, TNF‐α and IL‐1β (H) of H9C2 cells, N = 12. Western blots images (I) and analysis (J) of TLR4, TRAF6 and p‐NF‐κB from different groups in H9C2 cells, N = 3. *P < .05, **P < .01, **P < .01

Firstly, we investigated the anti‐inflammatory effects of danshen on macrophages, and LPS‐induced RAW264.7 cell model was built. The CCK8 results showed that co‐treatment of RAW264.7 cells with danshen below 1200 μg/mL was non‐toxic (Figure 5A), and danshen could obviously decrease the level of TNF‐α and NO (Figure 5B,C). We also found that LPS stimulation promoted p‐NF‐κB expression and nuclear translocation, as compared to the untreated cells, while danshen treatment could reverse the changes (Figure 5D). These in vitro results jointly confirmed that the anti‐inflammatory effect of danshen was partly exerted by inhibiting activity of macrophages.

We further explored the anti‐inflammatory effects of danshen on cardiomyocytes. The macrophage condition media (CM)‐induced inflammatory H9C2 cell model was established in our previous study.^15^ The CCK8 results showed that pre‐ and co‐treatment of H9C2 cells with danshen at the concentration of 600‐1200 μg/mL significantly increased the cell viability (Figure 5E) and reduced the release of LDH, TNF‐α and IL‐1β (Figure 5F,H). Furthermore, Western blots results showed that the expressions of TLR4, TRAF6 and p‐NF‐κB were down‐regulated by danshen treatment, as compared to CM‐stimulated H9C2 cells (Figure 5I). Collectively, these indicated that danshen exerted a good efficacy against inflammatory response and regulated TLR4 signalling pathway in cardiomyocytes.

### 3.6 TLR4 may mediate the inflammatory signalling pathway in cardiomyocytes

Intriguingly, TLR4 siRNA also increased the cell viability (Figure 5G) and attenuated release of LDH, TNF‐α and IL‐1β, respectively (Figure 5H), as compared to the CM group. Furthermore, Western blots results showed that the expressions of TLR4, TRAF6 and p‐NF‐κB were down‐regulated by TLR4 siRNA, as compared to CM‐stimulated H9C2 cells (Figure 5I). Collectively, these indicated that TLR4 signalling cascades may be an attractive target against inflammatory injury in cardiomyocytes.

### 3.7 Danshen ameliorates inflammatory injury by inhibiting TLR4‐TRAF6‐NF‐κB signalling pathway MD2/TLR4‐MyD88 complex formation

**Figure 6:**
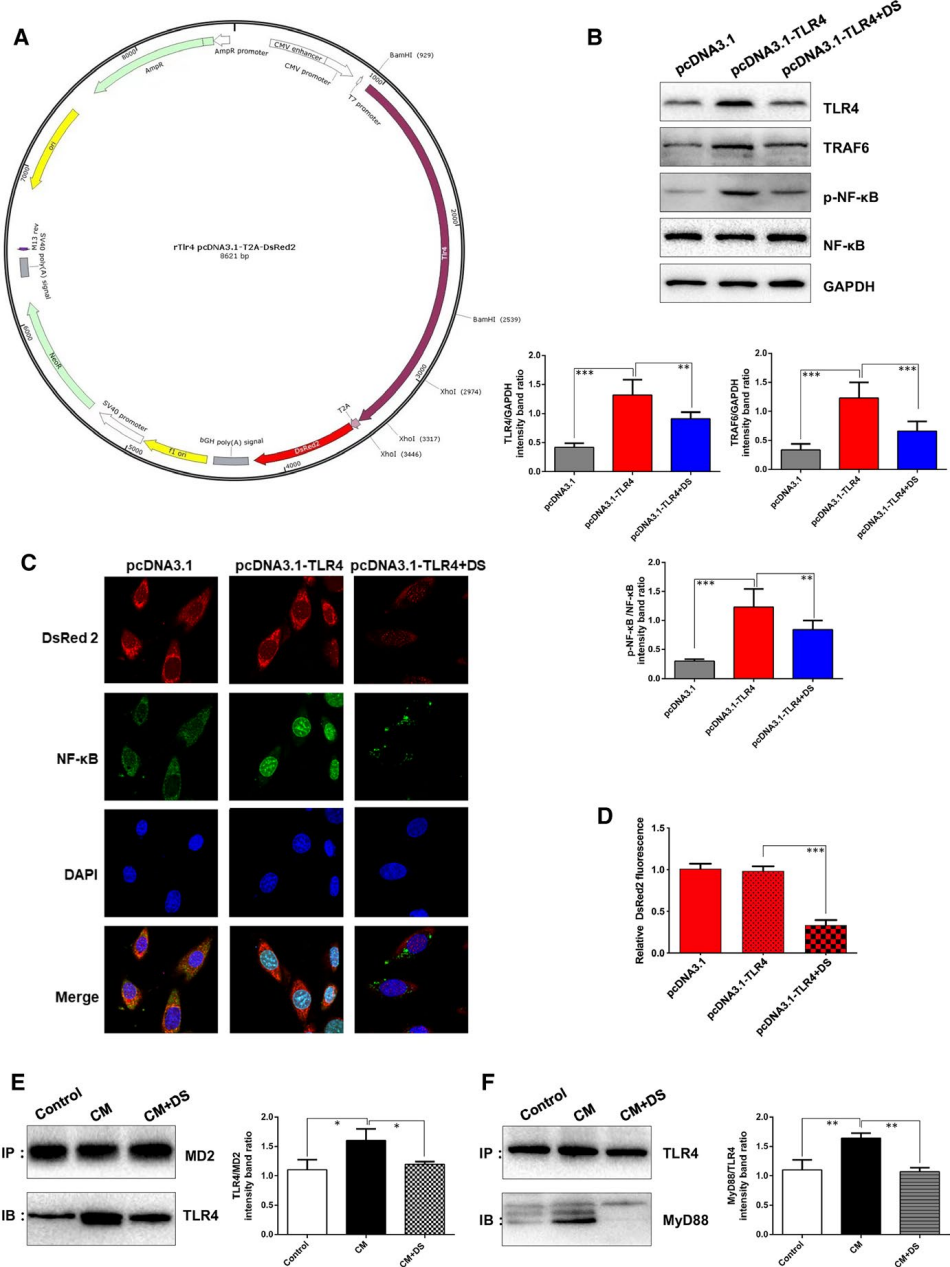
Danshen is shown to act on TLR4‐TRAF6‐NF‐κB signalling pathway and MD2/TLR4‐MyD88 complex formation. (A) The structure of DsRed2‐pcDNA3.1‐TLR4. (B) Western blots images and analysis of TLR4, TRAF6 and p‐NF‐κB from different groups, N = 3. (C) Cardiomyocytes were transfected with DsRed2‐pcDNA3.1‐TLR4 to monitor the expression of TLR4. DsRed2‐pcDNA3.1 transfected cell was set as a negative control. Representative DsRed2 fluorescence and immunofluorescence staining of NF‐κB were shown from different groups, N = 15. (D) The quantification of DsRed2 fluorescence, N = 15. The binding of MD2 and TLR4 (E) and the interaction of TLR4 and MyD88 (F) were detected by immunoprecipitation assay. **P < .01, **P < .01

To determine whether TLR4 was a direct target of danshen on regulating inflammation, we successfully overexpressed TLR4 by transfection with pcDNA3.1‐TLR4 plasmid (Figure 6A). Western blots revealed that TLR4, TFAF6 and activated NF‐κB were dramatically increased when compared with the pcDNA3.1 negative group, suggesting that TLR4 could trigger the NF‐κB‐activated inflammation in a cascade regulation (*P* < 0.001 vs pcDNA3.1 negative group; Figure 6B). Besides, danshen could inhibit the expressions of TLR4, TFAF6 and activated NF‐κB, respectively, as compared to pcDNA3.1‐TLR4 group (Figure 6B,C). Furthermore, immunofluorescence results showed that TLR4 overexpression (cells were transfected with DsRed2‐pcDNA3.1‐TLR4) significantly increased the nuclear translocation of NF‐κB, as compared with DsRed2‐pcDNA3.1 transfected cell (Figure 6C). While danshen treatment inhibited NF‐κB activation, leading to decreased nuclear content (Figure 6C). Intriguingly, the lower red fluorescence indicated a reduction of exogenous TLR4 expression in danshen‐treated cells (Figure 6D), suggesting that danshen directly contributes to the TLR4 protein degradation or inhibition of promoter. In the current study, CMV promoter and T7 promoter were applied in TLR4 plasmid. And we will furtherly investigate whether danshen directly contributes to the TLR4 protein degradation or inhibition of promoters in future. Taken together, these data indicated that danshen could act on TLR4‐TRAF6‐NF‐κB cascade signalling pathway to resist inflammation.

Furthermore, the binding of MD2 to TLR4 was explored. Meanwhile, TLR4‐MyD88 interaction was also investigated by co‐immunoprecipitation. Compared with the control group, CM stimulation increased the binding of MD2 to TLR4 and the interaction of TLR4 and MyD88, whereas danshen treatment significantly attenuated CM‐induced formation of MD2/TLR4‐MyD88 complex (Figure 6E,F).

## 4 DISCUSSION

Experimental work and clinical trials both showed that danshen have potent cardioprotective effects against HF.^11^, ^20^, ^21^, ^22^ However, the protective mechanisms of danshen need to be fully elucidated. Based on transcriptome technique, we explored the regulatory effects of danshen on AMI‐induced HF by in vivo and in vitro experiments. Our main findings are as below: (1) Danshen with different dosages attenuated cardiac dysfunction and reduced inflammatory response in AMI‐induced HF rats. (2) Transcriptome analysis and validation results revealed that TLR4 signalling pathway was involved in the anti‐inflammation effects of danshen. (3) Knock‐down and overexpression of TLR4 furtherly confirmed that danshen ameliorated inflammatory injury via TLR4‐TRAF6‐NF‐κB signalling pathway. (4) Danshen inhibited the formation of MD2/TLR4‐MyD88 complex.

Approximately 50% of all HF patients suffer from declined ejection fraction (typically considered as EF <40%).^23^ This form of HF is characterized by the correlation between adverse clinical events and raised serum/myocardial concentrations of pro‐inflammatory cytokines.^24^, ^25^ With respect to the pathological mechanism, inflammation has been documented as a critical event in AMI‐induced HF.^4^, ^25^ Following myocardial infarction, the primary objective of the inflammatory response in the heart is to repair tissue injury, thereby allowing the heart to adapt to the bad conditions in the short‐term and ultimately return to homeostasis and recover cardiovascular function in the long‐term.^4^, ^26^ However, under a sustaining abnormal state, a chronic inflammatory condition persists in cardiac tissue, furtherly causes the deleterious influence on cardiomyocytes and the extracellular matrix, eventually contributes to HF occurrence and development.^27^, ^28^ Given that inflammation itself is diverse and complex, anti‐inflammatory strategies have offered disappointing results so far. In this regard, a number of researches have shown the indispensable role of TLRs in several cardiovascular pathological mechanism, suggesting that the modulation of TLRs signalling pathway will be of great significance for cure.^8^, ^29^ All known human TLRs in the heart have been detected, and foremost is TLR4, whose level is the highest compared to other TLRs in the heart.^8^, ^30^ What's more, TLR4 plays a major role in myocardial inflammation, including MI, myocardial I/R injury, myocarditis, aortic valve diseases, hypertension, atherosclerosis and HF.^29^ Pharmacological intervention using TLR4 antagonists has been a challenging approach for the last two decades; nevertheless, these candidates failed in the stages of clinical trials, and therefore, discovery of new TLR4 modulators is much anticipated.^31^, ^32^

Medicinal herbal products have been applied for healthcare in Asia for over thousands of years. Recently, numerous researches have demonstrated that Chinese herbal medicines and bioactive phytochemicals can be promising cardioprotective therapies.^33^, ^34^ Danshen, the dried root or rhizome of Salvia miltiorrhiza Bge, is an ancient antipyretic traditional Chinese medicine and mostly used to ameliorate blood circulation and dispel blood stasis.^21^ Besides, it also plays beneficial roles in improving cardiovascular diseases, such as myocardial ischaemia, atherosclerosis, hypertension and HF.^20^ Data about clinical statistics of hospitalized patients and outpatients with cardiovascular diseases have demonstrated that danshen was top‐ranked (63.10% and 17.1%, respectively) out of all the clinical administered drugs in Beijing, Tianjin and Taiwan.^12^ These pharmacological activities are associated with inhibiting inflammation, attenuating post‐infarct remodelling, activating blood circulation, scavenging oxidative stress and improving angiogenesis via regulating multiple pathways. However, whether danshen could exhibit anti‐inflammatory effect against HF needs to be proved. Intriguingly, the results of our study showed that danshen treatment in different dosages improved LV function, mitigating pathological changes, inflammatory response including inflammatory cells infiltration and inflammatory cytokines releasing in AMI‐induced HF rats. Then, transcriptome technique was carried out to elucidate potential mechanisms of danshen. The KEGG pathway enrichment analysis as well as the functional analysis indicated that the differently expressed genes between danshen‐treated group and model group were involved in several pathways, primarily focused on the inflammation‐related TLRs signalling pathway. Based on differentially expressed genes in TLRs signalling pathway, we validated HF‐related gene Tlr4, Irf5, Cd86, Tlr9, Tlr2, Spp1, Cd80 and Tlr8. Of the eight genes, Tlr4, Tlr9 and Tlr8 are involved in the progression of HF^7^, ^35^, ^36^; silencing of the transcription factor Irf5 may be used to ameliorate HF^37^; immune response‐related genes CD80 and CD86 promise to be effective new immunotherapeutic targets in inflammation related to heart^38^; MI could induce strong transcriptional activation of Spp1.^39^ The regulation of danshen on these genes proved that danshen has reliable efficacy in treating HF. Given the significance of TLR4 in myocardial inflammation, we furtherly focused on the downstream molecular pathway of TLR4 signalling. In vivo Western blots showed that the levels of MyD88, TRAF6, p‐IκB, p‐p38 MAPK and p‐NF‐κB were down‐regulated by danshen treatment, which indicating that the anti‐inflammation effect of danshen may exert through the MyD88‐dependent TLR4‐TRAF6‐NF‐κB pathway.

In addition to immune cells, TLR4 has been detected in various cell types including cardiomyocytes,^40^ whereas the definite molecular mechanism needs to be fully elucidated in cardiomyocytes. We conducted a CM‐induced inflammatory H9C2 cell model, and TLR4 siRNA was utilized. Results demonstrated that knock‐down of TLR4 has a good effect against inflammatory injury, indicating TLR4 signalling pathway holds a great value against inflammation in stressed cardiomyocytes. Besides, in vitro results also showed that danshen protected against inflammatory injury and inhibited the expressions of TLR4, TRAF6 and activated NF‐κB. To further confirm whether danshen alleviates inflammation via regulating TLR4 signalling pathway directly, overexpression of TLR4 in H9C2 cell was utilized by pcDNA3.1 transient transfection carrying DsRed2. Overexpression of TLR4 impressively increased the levels of TLR4 and TRAF6, induced NF‐κB activation and nuclear translocation, indicating that TLR4 can induce NF‐κB‐mediated inflammation through Myd88‐dependent TRAF6 signalling pathway. Danshen treatment could down‐regulate TLR4, TRAF6 and activated NF‐κB, respectively, demonstrating that danshen exhibited anti‐inflammation effects through TLR4‐TRAF6‐NF‐κB signalling pathway. More importantly, by utilizing co‐immunoprecipitation, danshen was proved to suppress MD2/TLR4 complex formation and the interaction of TLR4 and MyD88, which consequently regulating the downstream pathways. Intriguingly, results in Figure 4 and Figure 5 showed that danshen decreased TLR4 expression both in vitro and in vivo. Marylene Y. et al demonstrated that the involvement of downstream MAPK signalling in modulation of TLR expression is critical,^41^ we assumed that the down‐regulatory effect of danshen on TLR4 expression may be due to the positive control feedback of MAPK signalling on TLR4 transcriptional regulation. More experiments are needed to confirm the assumption. In addition, it has been well established that in HF patients, down‐regulating pro‐inflammatory cytokines of macrophages suggest a direct cardioprotective effect.^42^ Intriguingly, our in vitro results also showed that secretion of pro‐inflammatory cytokines and activation of NF‐κB in macrophages were also inhibited by danshen. Collectively, danshen may serve as anti‐inflammation agents to prevent the development of HF.

Majority of clinical trials targeting aspects of inflammation in patients with heart failure have been largely negative,^43^ novel pathological mechanism and the drug targets of heart failure need to be further explored. This experiment elucidated the critical role of MD2/TLR4‐MyD88 complex formation and TLR4‐TRAF6‐NF‐κB signalling pathway in HF. And by targeting this pathway, the anti‐inflammatory effect of danshen was confirmed and a significant cardioprotective effect was achieved, this will provide alternative therapeutic strategies for the treatment of HF, even other cardiovascular diseases with inflammatory response as the core pathological mechanism.

There are still some limitations in the current study. The dose of danshen is converted from the Danqi pill, and more concentrations should be included to make the study more comprehensive. Moreover, the major active components of danshen and their binding sites at a structural level in regulating MD2/TLR4‐MyD88 complex formation and signalling are still unclear and more studies should be conducted to further screen and validate the active ingredients.

## 5 CONCLUSIONS

**Figure 7:**
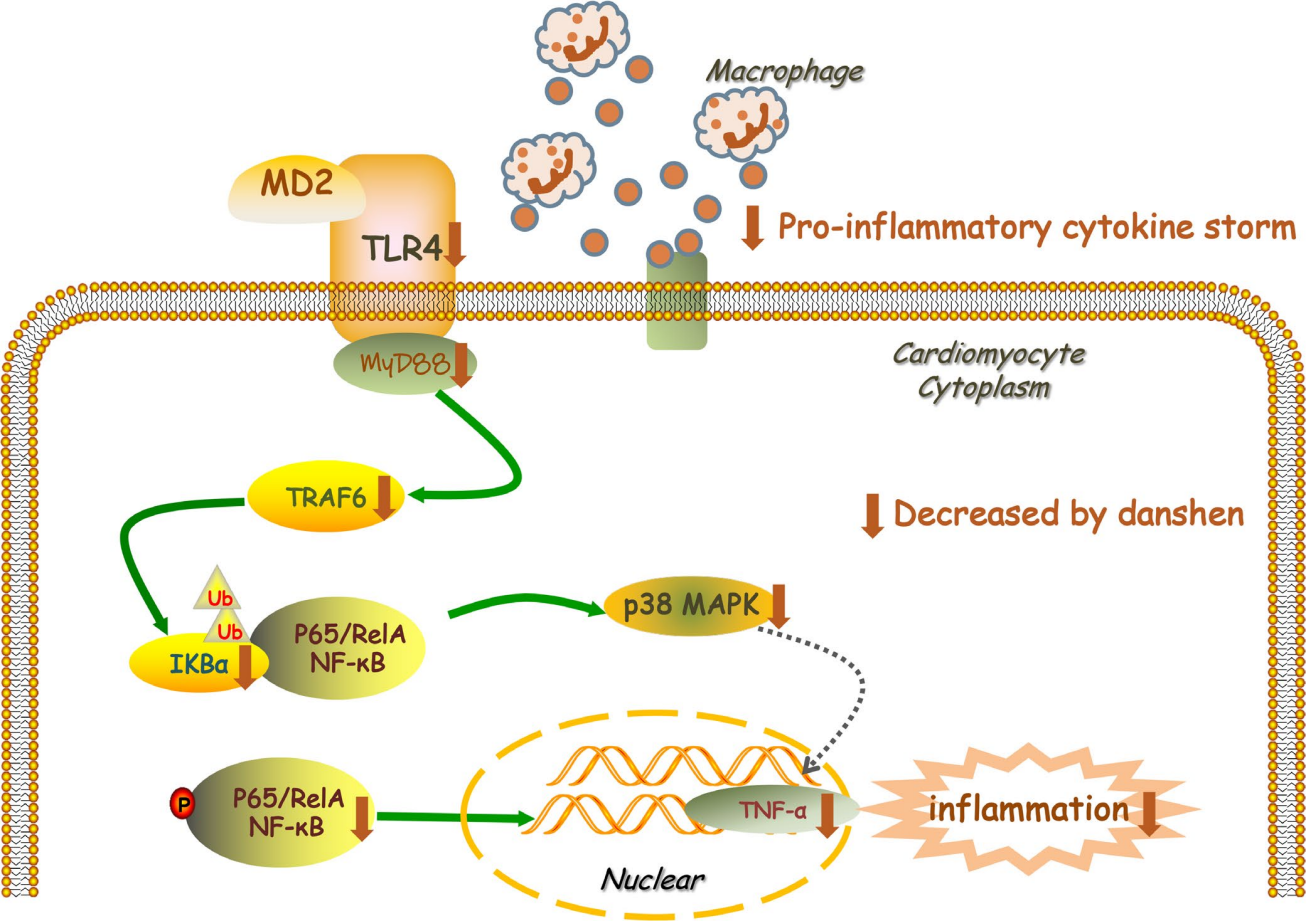
Potential mechanism of danshen on heart failure is mediated by MyD88‐dependent TLR4‐TRAF6‐NF‐κB inflammation pathway’

Our research evaluated the anti‐inflammation effects of danshen in vivo and in vitro. Results showed that danshen ameliorates inflammatory injury via MD2/TLR4‐MyD88 complex formation and TLR4‐TRAF6‐NF‐κB signalling pathway in AMI‐induced HF (Figure 7), providing further evidence that TLR4 signalling cascade is an attractive target against AMI‐induced HF. Besides, discrimination and quality assessment of danshen provided further interpretation of bioactive components. All in all, danshen promises to serve as a natural small molecule inhibitor for resisting inflammation in HF progression.

## CONFLICT OF INTEREST

The authors declare no conflict of interest.

## AUTHOR CONTRIBUTION

Xiaoping Wang: Writing‐original draft (lead); Writing‐review & editing (lead). Dongqing Guo: Writing‐review & editing (equal). Weili Li: Methodology (equal). Qian Zhang: Methodology (equal). Yanyan Jiang: Visualization (lead). Qiyan Wang: Writing‐review & editing (equal). Chun Li: Funding acquisition (equal). Qi Qiu: Funding acquisition (equal). Yong Wang: Funding acquisition (lead); Writing‐review & editing (equal).

## Supporting information

Table S1

Table S2

## Data Availability

The data sets used and/or analysed during the current study are available from the corresponding author on reasonable request.
